# 8-Prenylgenistein Isoflavone in Cheonggukjang Acts as a Novel AMPK Activator Attenuating Hepatic Steatosis by Enhancing the SIRT1-Mediated Pathway

**DOI:** 10.3390/ijms25179730

**Published:** 2024-09-08

**Authors:** Radha Arulkumar, Hee Jin Jung, Sang Gyun Noh, Hyun Woo Kim, Hae Young Chung

**Affiliations:** 1 Interdisciplinary Research Program of Bioinformatics and Longevity Science, Pusan National University, Busan 46241, Republic of Korea; rskrsk92@naver.com (S.G.N.); khw12424@naver.com (H.W.K.); 2 Department of Pharmacy, College of Pharmacy, Research Institute for Drug Development, Pusan National University, Busan 46241, Republic of Korea; hjjung2046@pusan.ac.kr

**Keywords:** fermented soybean, cheonggukjang, 8-prenylgenistein, hepatic steatosis, AMP-activated protein kinase, sirtuin 1

## Abstract

8-Prenylgenistein (8PG), a genistein derivative, is present in fermented soybeans (Glycine max), including cheonggukjang (CGJ), and exhibits osteoprotective, osteogenic, and antiadipogenic properties. However, the hepatoprotective effects of 8PG and its underlying molecular mechanisms remain largely unexplored. Here, we identified the high binding affinity of 8PG with AMP-activated protein kinase (AMPK) and sirtuin 1 (SIRT1), which acts as a potent AMPK activator that counteracts hepatic steatosis. Notably, 8PG exhibited better pharmacokinetics with greater absorption and higher plasma binding than the positive controls for the target proteins. Moreover, 8PG exerted non-carcinogenic activity in rats and significantly increased AMPK phosphorylation. Compound C, an AMPK inhibitor, did not antagonize 8PG-activated AMPK in HepG2 cells. 8PG significantly attenuated palmitate-induced lipid accumulation and enhanced phosphorylated AMPK and its downstream target, acetyl-CoA carboxylase. Further, 8PG activated nuclear SIRT1 at the protein level, which promoted fatty acid oxidation in palmitate-treated HepG2 cells. Overall, 8PG acts as a potent AMPK activator, further attenuating hepatic steatosis via the SIRT1-mediated pathway and providing new avenues for dietary interventions to treat metabolic dysfunction-associated steatotic liver disease (MASLD).

## 1. Introduction

Hepatic steatosis is a hallmark of metabolic dysfunction-associated steatotic liver disease (MASLD), formerly referred to as nonalcoholic fatty liver disease (NAFLD), and is characterized by excessive accumulation of fat in the liver [1,2]. Hepatic steatosis is mainly caused by de novo lipogenesis and impaired fatty acid oxidation in the liver [3]. Hepatic steatosis has been linked to gene dysfunction, improper diet, aging, and a lack of physical and social activities [4]. Aging also contributes to MASLD in older people, especially in individuals over 60 years [5]. The prevalence of MASLD has been increasing over the years and is highest in Southeast Asia, with a prevalence of 32.87% in the Republic of Korea [6]. Several epidemiological studies have shown that visceral obesity is closely linked to cardiovascular risk factors, such as dyslipidemia, insulin resistance, and type 2 diabetes [7,8,9,10]. Owing to the side effects of modern medicines, identifying novel potential agents from dietary supplements could be an effective strategy for developing interventions against aging-associated liver diseases without adverse effects.

Cheonggukjang (CGJ), a popular fermented soybean product used as a major dietary source in the Republic of Korea, contains many bioactive constituents, including organic acids, amino acids, fatty acids, and volatile compounds [11]. CGJ exhibits various beneficial health effects on metabolic syndrome, including preventing obesity, and fat accumulation, maintaining serum lipid levels, and suppressing inflammation in the liver, kidney, and spleen [12,13]. CGJ also exhibits antioxidant, antimicrobial, anticancer, and antidiabetic activities [14,15]. The consumption of fermented soybeans is significantly correlated with a reduction in the occurrence of breast and prostate cancer [16]. Moreover, fermented soybean consumption has been reported to increase aglycone isoflavone content and bioavailability during soybean fermentation [17,18]. Genistein, a soy-derived phytoestrogen, has been extensively studied for over 60 years. Fermented soybeans contain several undiscovered compounds that are significant to pharmaceutical researchers.

Owing to the large number of conceivable compounds in CGJ, analyzing their potential functional properties remains challenging. Recent advancements in computational methodologies have helped elucidate the pharmacological effects of bioactive compounds in a cost-effective and time-efficient manner [19,20]. Evaluating the absorption, distribution, metabolism, and excretion (ADME) properties of compounds is crucial for drug development [21]. 8-Prenylgenistein (8PG), a prenylated genistein derivative, shares a similar chemical structure with its parent compound genistein and exerts a strong anti-adipogenic effect at minimal concentrations with high bioavailability [22]. Furthermore, 8-prenyldaidzein (8PD) and 8PG exert greater anti-inflammatory effects than their parent compounds, daidzein and genistein, respectively [23]. Therefore, combining computational analysis with experimental evaluation can accurately demonstrate the effects of derivatives and bioactive compounds.

In this study, we screened several active compounds in CGJ and selected 8PG, which had improved ADME properties, through computational analysis. 8PG, a genistein derivative, is abundant in fermented soybeans (CGJ); however, its hepatoprotective effects and underlying molecular mechanisms have not been investigated. Therefore, this study aimed to determine the effects of 8PG on hepatic steatosis using an integrated approach.

## 2. Results

### 2.1. Identification and Characterization of Bioactive Metabolites from Cheonggukjang (CGJ)

**Figure 1:**
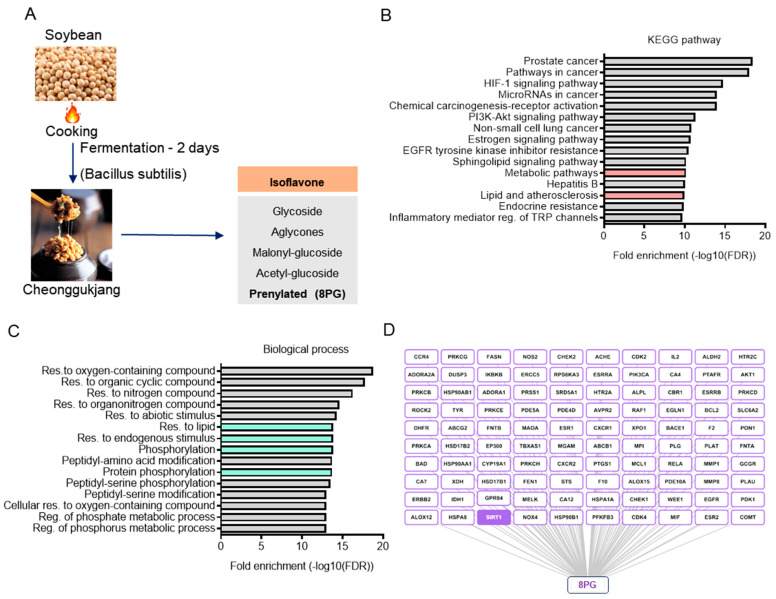
Identification, functional evaluation, and network pharmacology analysis of 8PG. (**A**) Methodology for preparing cheonggukjang (CGJ). (**B**,**C**) Enriched KEGG pathways and biological process of the overlapped targets of 8PG based on a ShinyGO (ver0.77) web server. (**D**) Target networks in 8PG. 8PG, 8-prenylgenistein; KEGG, Kyoto encyclopedia of genes and genomes; SIRT1, sirtuin 1.

CGJ is prepared via short-term fermentation with *Bacillus* species, predominantly *Bacillus subtilis,* and has significant amounts of sugar, amino acid, and isoflavonoid metabolites, including 8PG (Figure 1A and Supplementary Table S1) [24]. Recently, derivative compounds have gained attention because of their superior biological activities compared to those of their parent compounds. Thus, some isoflavone derivatives, specifically a class of flavonoids belonging to the family of polyphenolic compounds that have anti-inflammatory, antioxidant, and antimicrobial properties, in CGJ were screened [25,26,27,28]. Among the six isoflavones, 8PG exhibited the highest human intestinal absorption (HIA; 90.77%), plasma protein binding (98.93%), and non-carcinogenic activity (Supplementary Table S2), indicating superior pharmacokinetic (PK) values. P-glycoprotein (P-GP) is a member of the ATP-binding transmembrane glycoprotein family (ATP-binding cassette [ABC]), which can excrete drugs or other exogenous chemicals from cells [29]. As expected, 8PG was predicted to be a P-GP inhibitor that satisfied the ADMET and drug-likeness criteria, indicating its potential for further analysis. With a probability of more than 0.1, 100 targets of 8PG were obtained using Swiss target prediction version 2019 online tool.. Analysis of the KEGG pathway and biological processes of the 8PG target genes revealed that most were involved in signaling pathways related to lipid metabolism and regulation of post-translational modifications of proteins, such as phosphorylation (Figure 1B,C). Furthermore, SIRT1 was identified as a notable target of 8PG in its compound-target network (Figure 1D). These in silico data suggest that the pharmacological action of 8PG may involve the regulation of lipid metabolism, such as hepatic steatosis, by targeting AMPK, which is activated by phosphorylation, and SIRT1.

### 2.2. 8PG Has the Highest Affinity with AMPK and SIRT1 as Validated by Molecular Dynamics (MD) Simulation In Silico

**Figure 2:**
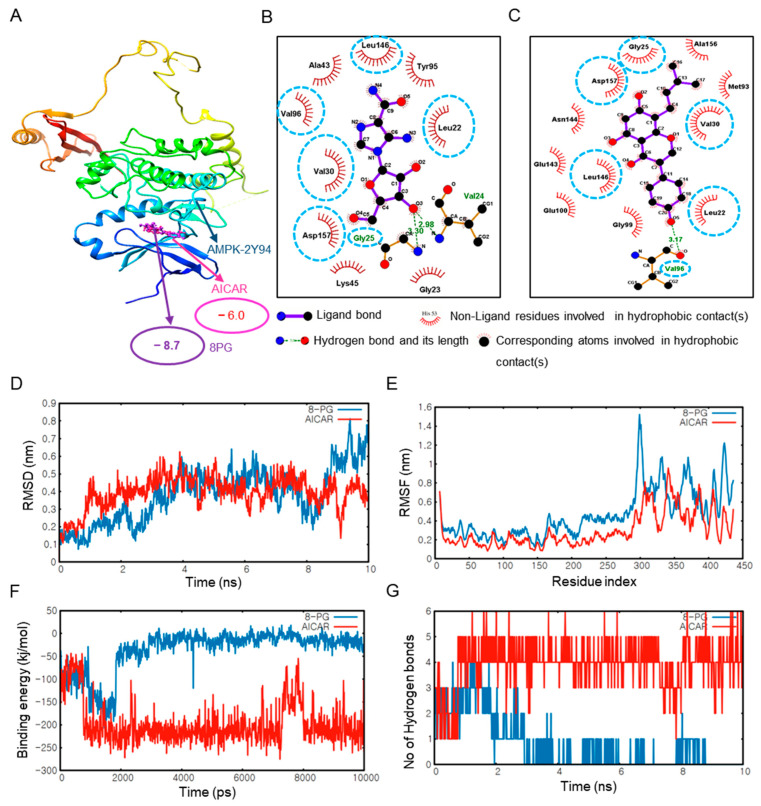
Molecular docking analysis of 8PG and AICAR with AMPK using AutoDock 4.2.6. (**A**) Binding interactions of the control, AICAR (pink), and 8PG (purple) with the AMPK protein. (**B**) Two-dimensional pharmacophore analysis between AMPK and the control compound, AICAR (pink box). (**C**) Two-dimensional pharmacophore analysis between AMPK and the active component, 8PG (purple box). The AutoDock 4 result was used for visualization and pharmacophore analysis. Commonly shared residues between AICAR and AMPK, and 8PG and AMPK were depicted by the cyan blue dotted circle. (**D**–**G**) Molecular dynamics simulation of 8PG and AMPK, and AICAR and AMPK. (**D**) RMSD plots between 8PG and AMPK, and AICAR and AMPK. (**E**) RMSF plots between 8PG and AMPK and AICAR and AMPK. (**F**) Binding interaction plots between 8PG and AMPK and AICAR and AMPK. (**G**) Number of hydrogen bonds between 8PG and AMPK and AICAR and AMPK at 10 ns. AICAR, 5-aminoimidazole-4-carboxamide ribonucleotide; 2D, two-dimensional; 8PG, 8-prenylgenistein; AMPK, adenosine 5′ monophosphate-activated protein kinase; ns, nanosecond; RMSD, root-mean-square deviation; RMSF, root-mean-square fluctuation.

**Figure 3:**
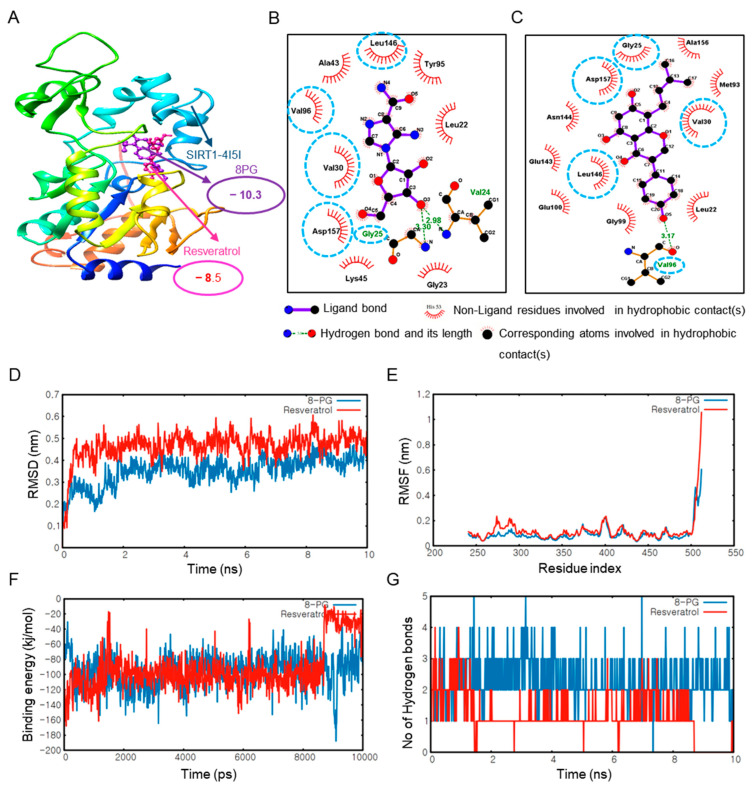
Molecular docking analysis of 8PG and resveratrol with SIRT1 using AutoDock 4.2.6. (**A**) Binding interactions of the control, resveratrol (pink), and 8PG (purple) with the SIRT1 protein; (**B**) Two-dimensional pharmacophore analysis between SIRT1 and the control compound, resveratrol (pink box); (**C**) Two-dimensional pharmacophore analysis between SIRT1 and the active component, 8PG (purple box). The AutoDock 4 result was used for visualization and pharmacophore analysis. Commonly shared residues between resveratrol and SIRT1 and 8PG and SIRT1, were rounded by the cyan blue dotted circle. (**D**–**G**) Molecular dynamics simulation of 8PG and resveratrol with SIRT1. (**D**) RMSD plots between 8PG (purple) and SIRT1 and resveratrol (red) and SIRT1. (**E**) RMSF plots between 8PG and SIRT1 and resveratrol and SIRT1. (**F**) Binding interaction plots between 8PG and resveratrol with SIRT1. (**G**) Number of hydrogen bonds between 8PG and SIRT1 and resveratrol and SIRT1 at 10 ns. SIRT1, sirtuin 1; 2D, two-dimensional; 8PG, 8-prenylgenistein; ns, nanosecond; RMSD, root-mean-square deviation; RMSF, root-mean-square fluctuation.

As the potential targets of 8PG were predicted earlier, molecular docking of 8PG with targets, such as AMPK and SIRT1, was performed to predict the binding affinity relative to that of the positive control. Protein and ligand interactions are shown in Figure 2 and Figure 3, respectively. Compared to the control compounds, AICAR (AMPK) and resveratrol (SIRT1), 8PG had the highest binding energy score with ligands (Figure 2 and Figure 3). Moreover, 8PG and AMPK produced a compound with the lowest binding energy of −8.7 kcal/mol than other isoflavones, including their parental compound, genistein (Supplementary Table S3). In contrast, a value of −10.3 kcal/mol was obtained with SIRT1 (Table 1 and Table 2, and Supplementary Table S4), indicating that 8PG binds more strongly to AMPK and SIRT1 than to the control compounds, AICAR, and resveratrol. Furthermore, based on the 8PG-AMPK binding residues within AMPK, several residues, including LEU22, GLY25, VAL30, VAL 96, LEU146, and ASP 157, which shared common interactions, were identified (Figure 2B,C). Similarly, 8PG-SIRT1, GLY25, VAL30, VAL96, LEU146, and ASP157 residues commonly shared these interactions (Figure 3B,C).

The MD simulation technique helps mimic the conformational changes of a protein-ligand system over time. Here, the structural stability of AMPK and SIRT1 with 8PG, along with the control compounds, AICAR, and resveratrol, was evaluated based on their RMSD plots (Figure 2D and Figure 3D). The RMSD plots show that 8PG had lower fluctuations with AMPK (0.1–0.8 nm value) and SIRT1 (0.1–0.4 nm value) than the control within 10 ns. 8PG with the AMPK and SIRT1 targets attained equilibrium quickly compared with the controls. Furthermore, the average RMSD values imply less structural deviation in 8PG, resulting in improved structural stability of the complex. The structural flexibility of AMPK- and SIRT1-8PG compared with the control complex was assessed based on their per-residue RMSF plots (Figure 2E and Figure 3E) considering the Cα-atomic fluctuations. The molecular docking results were validated by measuring the interaction energies of the MD trajectories. The interaction energy values of 8PG with AMPK and SIRT indicated active interactions resulting in the formation of a stable complex (Figure 2F and Figure 3F). The number of H bonds formed between 8PG, AMPK, and SIRT1 during MD simulation was also calculated (Figure 2G and Figure 3G). More intermolecular H-bonds in the 8PG complex structure may help maintain its rigidity and participate in hydrogen bonding with the solvent, enabling more flexibility. 8PG formed a greater number of H bonds with AMPK and SIRT1 than the control, which exhibited fewer intermolecular H bonds. In addition, 8PG, which is predicted to be a P-GP inhibitor but not AICAR and resveratrol, had stronger absorption and higher plasma protein binding values than AICAR, the control compounds for AMPK (Table 3). Notably, resveratrol (the SIRT1 control) may be carcinogenic. Collectively, 8PG had stronger binding interaction and more negative interaction energy with AMPK and SIRT1, which confirms its potential as a candidate in silico.

### 2.3. 8PG Acts as an Activator of AMPK and Attenuates Lipid Accumulation in HepG2 Cells

**Figure 4:**
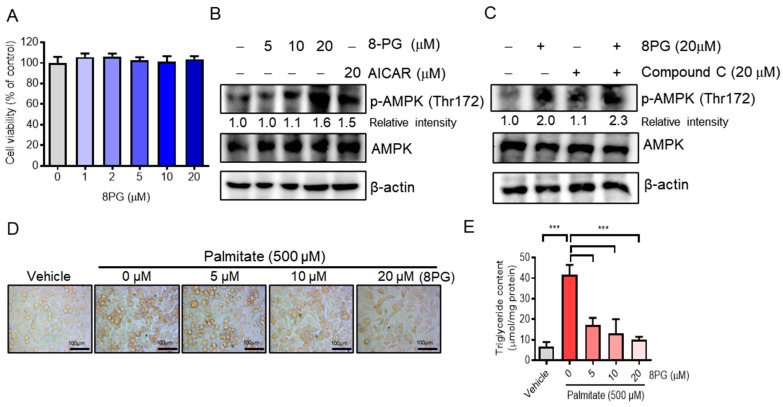
Effect of 8PG on AMPK activation and lipid accumulation in palmitate-treated HepG2 cells. (**A**) HepG2 cells were treated with different concentrations of 8PG for 24 h, and cell viability was determined using the Ez-Cytox assay. (**B**) HepG2 cells were treated with AICAR (20 µM) and 8PG (5, 10, and 20 µM) for 24 h. Protein expression of phosphorylated AMPK was analyzed using Western blotting. (**C**) HepG2 cells were treated with Compound C (20 µM) and 8PG (20 µM) for 24 h. Protein expression of phosphorylated AMPK was analyzed using Western blotting. (**D**) HepG2 cells were treated with palmitate (500 µM) and/or 8PG (5, 10, and 20 µM) for 24 h. Lipid accumulation was determined via ORO staining. Images of cells were captured at 200× magnification. (**E**) Measurement of intracellular TG contents. Data are presented as mean ± SD of three independent experiments (*** *p* < 0.001). 8PG, 8-prenylgenistein; AMPK, adenosine 5′ monophosphate-activated protein kinase; ORO, Oil Red O; SD, standard deviation; TG, triglyceride.

First, a cytotoxicity assay was performed in 8PG-treated HepG2 cells in vitro to confirm the in silico data. We found that up to 20 μM 8PG did not exhibit significant cytotoxic effects on HepG2 cells (Figure 4A). AMPK is a master regulator of metabolism, and its activation promotes fatty acid oxidation and restricts fatty acid synthesis [30,31]. To determine whether 8PG activates AMPK, HepG2 cells were treated with 8PG in vitro. 8PG was found to significantly increase AMPK phosphorylation in a dose-dependent manner in HepG2 cells, similar to AICAR (an AMPK activator) (Figure 4B). Moreover, compound C, an AMPK inhibitor, did not antagonize the 8PG-activated AMPK in HepG2 cells (Figure 4C), suggesting that 8PG is a novel AMPK activator. Oil Red O staining confirmed that 8PG significantly decreased lipid droplets (Figure 4D) and cellular triglyceride content in palmitate-treated HepG2 cells (Figure 4E). These findings suggest that 8PG attenuated palmitate-induced lipid accumulation in HepG2 cells.

### 2.4. 8PG Mitigates Fatty Acid Synthesis and Enhances β-Oxidation by Activating the SIRT1-Mediated Pathway in HepG2 Cells

**Figure 5:**
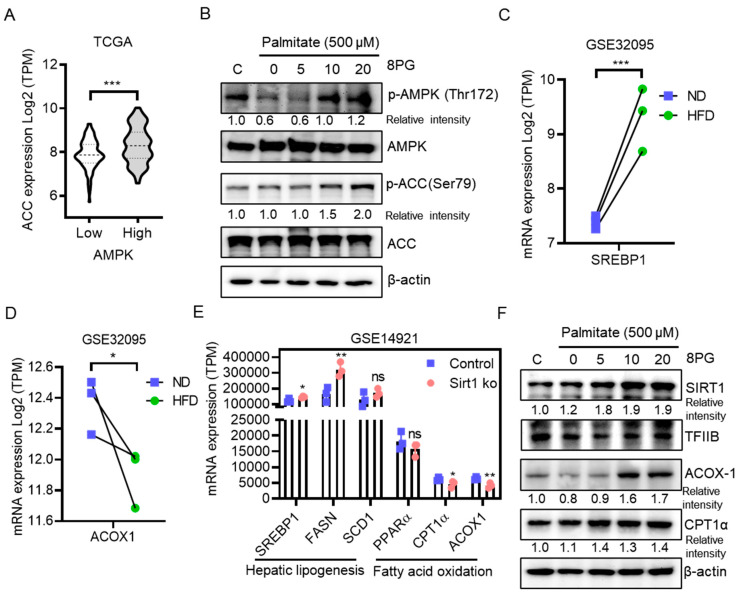
Effect of 8PG on AMPK activation and SIRT1-mediated lipogenesis and fatty acid oxidation in palmitate-treated HepG2 cells. (**A**) Samples with the highest AMPK expression and samples with the lowest AMPK expression were evaluated to determine the ACC levels in the liver tissue of healthy participants (n = 215) using (GENT2) (http://gent2.appex.kr/gent2/, accessed on 18 January 2024) databases. The data are expressed as mean ± standard deviation (SD), and asterisks (*) indicate significant differences between groups (*** *p* < 0.001). (**B**) Protein expression levels of phosphorylated AMPK, and ACC, in palmitate (500 µM)-treated HepG2 cells were analyzed using Western blotting. (**C**,**D**) mRNA expression of SREBP-1 and ACOX1 in the liver tissue of ND- and HFD-fed mice were obtained from the GSE32095 dataset (ND, n = 3; HFD, n = 3). Asterisks (*) indicate significant differences (*** *p* < 0.001) compared with the normal diet group. (**E**) mRNA expression of SIRT1-linked hepatic lipogenesis and fatty acid oxidation genes in the liver of control and SIRT1 knockout mice were obtained from the GSE14921 dataset (Control, n = 3; SIRT1 ko, n = 3). Asterisks (*) indicate significant differences (* *p* < 0.05 and ** *p* < 0.01) compared with the control group. (**F**) Protein expression levels of, ACOX1, CPT1α, and nuclear expression levels of SIRT1 in palmitate (500 µM)-treated HepG2 cells were analyzed using Western blotting. TFIIB was used as a loading control for the nuclear lysates. 8PG, 8-prenylgenistein; ACC, acetyl-CoA carboxylase; ACOX1, acyl-CoA oxidase-1; AMPK, adenosine 5′ monophosphate-activated protein kinase; CPT1α, carnitine palmitoyltransferase I alpha; FAS, fatty Acid Synthase; HFD, high-fat diet, ND, normal diet, SD, standard deviation; SIRT1, sirtuin 1; SREBP-1, sterol regulatory element-binding protein 1, TCGA, The Cancer Genome Atlas Program, TFIIB, transcription factor II b.

To further elucidate the molecular mechanisms of 8PG activation of AMPK and inhibition of lipid accumulation in HepG2 cells, the expression levels of genes involved in lipogenesis and fatty acid β-oxidation were analyzed in palmitate-treated HepG2 cells and publicly available transcriptome datasets (GSE32095 and GSE1491). According to a previous study, ACC is a downstream target of AMPK and is associated with reduced lipid biosynthesis [32]. Furthermore, clinical transcriptomic analysis of non-diseased liver tissues from The Cancer Genome Atlas Program (TCGA) revealed that ACC expression is positively associated with AMPK levels (Figure 5A). 8PG significantly increased the protein levels of phosphorylated AMPK and subsequently phosphorylated ACC in palmitate-treated HepG2 cells (Figure 5B), thereby inhibiting fatty acid synthesis. Consistent with these data, a high-fat diet (HFD) increased the expression of the lipogenesis gene, sterol regulatory element-binding protein 1 (SREBP-1), and decreased the expression of the fatty acid oxidation gene, acyl-CoA oxidase 1 (ACOX1), compared with a normal diet (ND) in mouse liver (Figure 5C,D).

SIRT1 is an NAD+-dependent type III nuclear deacetylase that acts as a metabolic sensor of NAD+ and regulates various cellular metabolic pathways [33]. Moreover, activators of SIRT1 play an important role in maintaining lipid and glucose homeostasis and insulin sensitivity, which is mediated by mitochondrial biogenesis and β-oxidation [34,35]. Moreover, the inactivation of SIRT1 was linked with the activation of lipogenesis (SREBP-1 and fatty acid synthase [FASN])- and inhibition of the beta-oxidation-associated genes (Carnitine Palmitoyltransferase 1α [*CPT1α*] and *ACOX1*) in mouse liver (Figure 5E). To confirm the activation of SIRT1 in palmitate-treated HepG2 cells, nuclear extracts were examined using immunoblotting. The expression of nuclear SIRT1 at the protein level increased in palmitate-treated HepG2 cells (Figure 5F). Further, 8PG increased the protein levels of fatty acid β-oxidation genes, such as *ACOX1* and *CPT1α*, in palmitate-treated HepG2 cells (Figure 5F). Collectively, these findings indicate that 8PG acts as an activator of AMPK and is associated with the reduction of lipid accumulation and stimulation of the fatty acid β-oxidation in HepG2 cells in vitro, thereby attenuating hepatic steatosis.

## 3. Discussion

Aging is the most common risk factor for MASLD [5] and is a major socioeconomic burden worldwide. Hepatic steatosis is the first stage in the development of MASLD and is associated with various metabolic disorders, including obesity and insulin resistance [3,7]. Patients with MASLD are more susceptible to the development of cirrhosis and hepatocellular carcinoma [36,37]. Thus, identifying effective compounds in regular diets that inhibit lipid accumulation and enhance fatty acid oxidation is vital for preventing MASLD. CGJ is a traditional Korean food made from fermented soybeans that contains high levels of aglycone isoflavone, which increases its bioavailability [17,18]. Through similar structure selection, ADMET prediction, network pharmacology, docking validation, and activity evaluation, biologically active substances, such as 8PG, in short-term fermented soybean products (CGJ), were screened to further demonstrate that 8PG acts as a novel activator of AMPK and to determine its anti-hepatic steatosis effect on HepG2 cells.

In silico KEGG pathway enrichment analysis and biological processes of the 8PG target genes revealed that its targets were mainly involved in lipid metabolism-associated signaling pathways and regulation of post-translational modifications of proteins, such as phosphorylation (Figure 1B,C). AMPK is a well-known regulator of metabolism that can stimulate fatty acid oxidation and restrict fatty acid synthesis [30,38]. The molecular docking results revealed that 8PG had the highest affinity for AMPK and SIRT1 among the predicted targets in the PPI network analysis (Figure 1D). Furthermore, 8PG exhibited better drug-likeness and ADMET properties than the AMPK (AICAR) and SIRT1 (resveratrol) agonists. Notably, 8PG had a higher binding interaction energy and more negative interaction energy, suggesting that these proteins could be potential candidates for 8PG. To validate this hypothesis, in vitro cell culture experiments were performed using HepG2 cells. In this study, 8PG significantly induced AMPK phosphorylation in a dose-dependent manner. Notably, compound C did not antagonize 8PG-activated AMPK in HepG2 cells, indicating that 8PG is a potent AMPK activator.

Hepatic steatosis is characterized by excess lipid accumulation in hepatocytes [39]. Analysis of a publicly available dataset revealed that the expression of the lipogenesis gene, *SREBP-1*, increased and that of the fatty acid oxidation gene, *ACOX1*, decreased in an HFD mouse model. In the present study, 8PG further decreased cellular triglyceride and lipid droplet levels in palmitate-treated HepG2 cells. AMPK phosphorylation is associated with the stimulation of ACC phosphorylation, which decreases lipid biosynthesis [32]. The next target of PPI network analysis, SIRT1, is a metabolic sensor of NAD+ that has been reported to be involved in various cellular metabolic processes [33] and plays a crucial role in hepatocellular lipid metabolism [35]. Further, AMPK raises NAD+ levels to activate SIRT1, a substrate for SIRT1 activity used by NAD+-dependent deacetylases [40].

**Figure 6:**
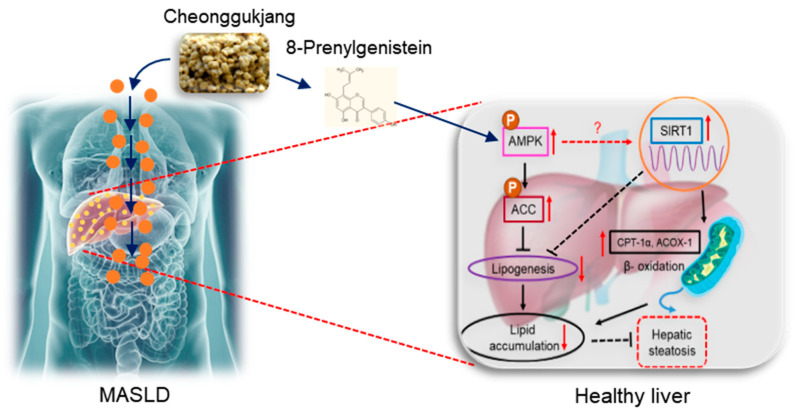
Schematic outlining the mechanism by which 8PG from CGJ mitigates hepatic steatosis. 8PG induced the activation of AMPK and its downstream target, ACC, and further increased the protein level of nuclear SIRT1, which is associated with the inhibition of hepatic lipogenesis and activation of fatty acid β-oxidation (CPT1α and ACOX1) in HepG2 cells, thereby counteracting hepatic steatosis. (↑ and ↓ indicate upregulation and downregulation, respectively). 8PG, 8-prenylgenistein; ACC, acetyl-CoA carboxylase; ACOX1, acyl-CoA oxidase-1; AMPK, adenosine 5′ monophosphate-activated protein kinase; CPT1α, carnitine palmitoyltransferase I alpha; FAS, fatty acid synthase; MASLD, metabolic dysfunction-associated steatotic liver disease; SIRT1, sirtuin 1; SREBP-1, sterol regulatory element-binding protein 1.

Based on the above evidence, 8PG can increase NAD+ synthesis, thereby activating SIRT1. It is necessary to examine the NAD+ levels in response to 8PG treatment. To evaluate the involvement of AMPK and SIRT1 activation in counteracting lipid accumulation in HepG2 cells, cells were exposed to 8PG after treatment with palmitate. Based on this assumption, 8PG significantly enhanced both phosphorylated AMPK and its downstream target, ACC, thereby counteracting palmitate-induced lipid accumulation. Furthermore, 8PG activated nuclear SIRT1 and fatty acid β-oxidation genes, such as *ACOX1* and *CPT1α*, at the protein level in palmitate-treated HepG2 cells (Figure 5). Functionally, inactivation of SIRT1 is linked to the activation of lipogenesis and inhibition of beta-oxidation pathways in the liver tissue of mice [41]. In addition, resveratrol, a SIRT1 enhancer, stimulated the expression of SIRT1 and phosphorylation of AMPK, resulting in the inhibition of lipid accumulation in the liver and recovery from hepatic steatosis in obese mice [42,43]. Notably, older animals tended to have lower AMPK activity and were associated with a lower metabolic rate than younger animals, indicating that a decrease in AMPK activity is linked to aging-related diseases, such as metabolic syndrome [44]. Hence, the activation of the AMPK signaling pathway by 8PG is an important mechanism for delaying aging. Together, these data indicate that 8PG acts as an AMPK activator and may attenuate lipid accumulation in hepatocytes by enhancing the SIRT1-mediated pathway (Figure 6).

## 4. Materials and Methods

### 4.1. Selection of Active Metabolites in Cheonggukjang (CGJ)

**Figure 7:**
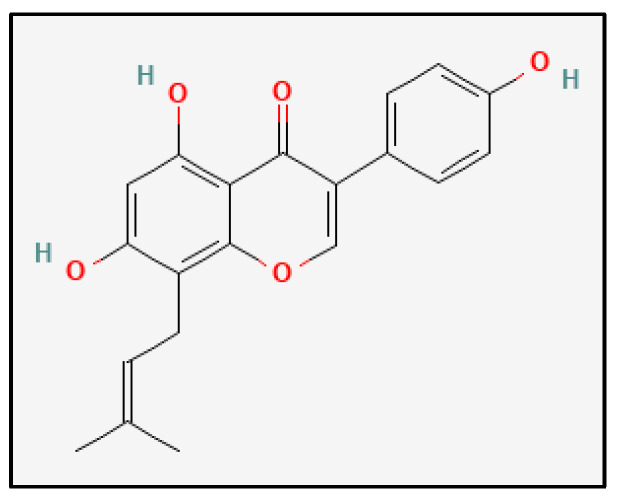
Chemical structure of 8-prenylgenistein (8PG).

CGJ contains several active metabolites, including isoflavone glycosides [24]. The common isoflavone genistein and its derivative 8PG were extracted and subjected to molecular docking and structural similarity analysis. In silico tests were conducted using the 3D chemical structure of 8PG and the crystalline forms of its receptors [AMPK, PDB ID: 2Y94 [45]; Sirtuin 1 (SIRT1), PDB ID: 4I5I [46]. Proteins and ligands were generated using UCSF Chimera software version 1.14 [47]. Using Marvin Sketch v17.1.30 (https://docs.chemaxon.com/display/lts-europium/marvinsketch-downloads.md/, accessed on 8 July 2021), the ligands were retrieved from the PubChem database (Figure 7) [48].

### 4.2. Network Pharmacology Analysis

The probable targets from the Homo sapiens species were retrieved using the target prediction computational tool, Swiss Target Prediction (http://www.swisstargetprediction.ch, accessed on 18 January 2024) [49]. Cytoscape software V.3.2.1 generated and viewed the predicted target networks and protein-protein interactions (PPIs) [50]. Using the default configuration with a “degree” value, network analysis was conducted. All proteins and genes underwent pathway enrichment analysis (KEGG analysis and biological processes) with ShinyGO (ver0.77) (http://bioinformatics.sdstate.edu/go/, accessed on 5 September 2021) [51].

### 4.3. Absorption, Distribution, Metabolism, Excretion, and Toxicity (ADMET) Property Prediction

Drug development and precise drug candidate prediction depend on the ability to predict absorption, distribution, metabolism, excretion, and toxicity, or ADMET. Because in silico methods can forecast the AD-MET profiles of chemically formulated and environmentally friendly next-generation medications early, they have transformed the management of the disease [52]. Orally active medication candidates are deemed worthy if they can mostly fulfill Veber’s rule of three [53] and Lipinski’s rule of five [54]. The pharmacokinetic parameters of the compounds that underwent selective screening were estimated using the online software PreADMET (https://preadmet.bmdrc.kr/adme/, accessed on 25 August 2021) [55]. The compounds were then calculated and their compliance with standard ranges was verified.

### 4.4. Molecular Docking

An in silico molecular docking analysis was carried out to determine 8PG’s binding affinity for the target molecules. The optimum score option was validated using three different docking programs. Using the Auto Dock v.4.2.6 program, molecular docking for the set of optimized ligands was carried out [56,57]. A sophisticated angle optimization method is used by AutoDock Vina as part of the local optimization strategy [58]. A score of less than zero in the docking system denotes a strong binding affinity between the receptor and ligand molecules [59]. In addition, the LeDock program was employed since it possessed the highest sampling power and 80.8% accuracy for the optimal positions. Utilizing UCSF Chimera V. 1.14 (https://www.rbvi.ucsf.edu/chimera/, accessed on 18 August 2021), the optimal scoring ligand molecule for receptor binding was identified. The 2D interactions of the protein-ligand complex structure, counting of hydrogen bonds, hydrophobic interactions, and bond lengths were analyzed using LigPlot+ [60] to identify high-affinity ties.

### 4.5. Molecular Dynamics Simulations

The Gromacs 5.1.2 program was utilized to conduct molecular dynamics (MD) simulations of the SIRT1-8PG complex [61]. The SIRT1 [62,63,64] atomic drive field parameters were constructed using CHARMM36, an all-atom-driven lipid force field. Using an automated topology builder (ATB, https://atb.uq.edu.au/index.py/, accessed on 28 August 2021) [65], the topology of 8PG, or the control atomic drive field parameters, was retrieved from the Gro-mos54a7 force field. The files were then transformed into the GROMACS file format. Energy minimization was performed first. Canonical ensembles (NVT) and isobar isothermal ensembles (NPT) were then employed. Ten nanoseconds were allotted to the MD runs. Calculations were performed on binding energy, hydrogen bonds, root-mean-square deviation (RMSD), and root-mean-square fluctuation (RMSF) following the runs. The Gnuplot application was used to analyze these parameters [66].

### 4.6. Public Datasets and Clinical Data Analysis

The public repository GEO database (https://www.ncbi.nlm.nih.gov/geo/, accessed on 18 January 2024) provided the mRNA expression of hepatic lipogenesis and fatty acid oxidation in datasets of high-fat diet-fed (GSE32095, n = 6) and SIRT1 knockout (GSE14921, n = 6) mice. In the livers of healthy individuals, the relationship between AMPK and acetyl-CoA carboxylase (ACC) was obtained and examined by the Gene Expression Database of Normal and Tumor Tissues 2 (GENT2, n = 215) (http://gent2.appex.kr/gent2/, accessed on 18 January 2024) [67]. Data from transcriptome analyses were received from the Geno-typeTissue Expression (GTEx) online portal (https://gtexportal.org/home/index.html/, accessed on 22 January 2024) for liver tissue of 226 normal persons across various age groups.

### 4.7. Reagents and Antibodies

8PG was purchased from ChemFaces (Wuhan, China). Dimethyl sulfoxide (DMSO), sodium palmitate, compound C, and Oil Red O (ORO) staining solutions were obtained from Sigma-Aldrich (St. Louis, MO, USA). Protein concentration was measured using bicinchoninic acid (BCA; Thermo Scientific, Waltham, MA, USA), with bovine serum albumin (BSA) as the standard. Polyvinylidene fluoride (PVDF) membranes were obtained from Millipore (Billerica, MA, USA). Primary antibodies against the following proteins were used for Western blot analysis: AMPK, p-AMPK (Thr172), ACC, p-ACC (Ser79), CPT1α, SIRT1, TFIIB, and β-actin; these antibodies were purchased from Santa Cruz Biotechnology, Inc. (Dallas, TX, USA).

### 4.8. Cell Culture and Treatment

HepG2, a human liver cancer cell line, was acquired from the American Type Culture Collection (ATCC, Manassas, VA, USA). The cells were cultivated in DMEM (Dulbecco’s Modified Eagle’s Medium) supplemented with 10% fetal bovine serum (Gibco, Carlsbad, CA, USA) at 37 °C in a 5% CO_2_ humidified environment. 8PG was purchased from ChemFaces (Wuhan, China). 8PG was dissolved in 100% DMSO. The final DMSO concentration did not exceed 0.1%.

### 4.9. Cell Viability

We determined the cell viability using the EZ-Cytox assay (Daeil Lab, Seoul, Republic of Korea). HepG2 cells (5 × 10^3^/well) were seeded in 96-well plates and incubated with 0–20 µM 8PG for 24 h. DMSO was used as vehicle control. The cells were incubated with EZ-Cytox solution following treatment. Tecan, Sunrise, USA manufactured a microplate reader that was used to detect absorbance at 450 nm. The percent inhibition due to 8PG was calculated using the following formula: inhibition (%) = [(OD (sample) − OD (control))/(OD (normal) − OD (control))] × 100. All assays were performed in triplicate and the results were averaged.

### 4.10. Preparation of Sodium Palmitate and Its Administration to HepG2 Cells

Palmitate was conjugated to fatty acid-free BSA (GenDEPOT, Barker, TX, USA) using a previously reported methodology [68]. Briefly, 69.6 mg of sodium palmitate was dissolved in 0.5 mL of 0.1 N sodium hydroxide at 70 °C to create a 500 mM stock solution. After the dissolution of palmitate, the stock solution was immediately added to serum-free DMEM (containing 5% fatty acid-free BSA) to create a 0.5 mM palmitate solution. The cells were pre-treated with various concentrations of 8PG (5–20 µM) for 24 h before exposure to palmitate (0.5 mM) for 24 h.

### 4.11. Oil Red O Staining

HepG2 cells were fixed with 4% paraformaldehyde and then incubated in 60% isopropanol and stained with Oil Red O (ORO) staining solution. The cells were washed several times with ddH_2_O to remove the excess stain. Images were captured using a microscope (Motic, Schertz, TX, USA).

### 4.12. Measurements of Intracellular Triglyceride Contents

An enzymatic colorimetric assay kit (Bio-Clinical System, Gyeonggi-do, Republic of Korea) was used to quantify intracellular triglyceride content in HepG2 cells after lysis with 1% Triton X-100 in PBS. The BCA method was used to determine protein concentrations, with BSA as the standard. A normalized calculation was performed to adjust intracellular triglyceride levels for cellular protein levels.

### 4.13. Western Blotting

We performed Western blotting as previously described [69]. Total cell lysates were prepared in gel-loading buffer (0.125 M Tris-HCl, pH 6.8, 4% SDS, 10% 2-mercaptoethanol, and 0.2% bromophenol blue). Then, the protein was separated by sodium dodecyl-sulfate polyacrylamide gel electrophoresis (SDS-PAGE) using 7–9% acrylamide gels and then transferred onto PVDF membranes (Millipore, Burlington, MA, USA) at 25 V for 10 min in a semi-dry transfer system (Bio-Rad Laboratories, Hercules, CA, USA). The membranes were immediately placed in blocking buffer (5% nonfat milk) containing 10 mM Tris (pH 7.5), 100 mM NaCl, and 0.1% Tween-20. The blots were then incubated at room temperature for 1 h. The membranes were incubated with appropriate specific primary antibodies at 4 °C overnight, followed by horseradish peroxidase-conjugated anti-mouse and anti-rabbit antibodies (1:5000) at 25 °C for 1 h (Santa Cruz Biotechnology, Dallas, TX, USA). Protein bands were visualized using the SuperSignal^®^ West Pico Chemiluminescent Substrate kit (Advansta, San Jose, CA, USA) and Davinch-Chemi^TM^ (Davinch-K, Seoul, Republic of Korea).

### 4.14. Statistical Analyses

We performed all statistical analyses with GraphPad Prism 8.02 (GraphPad Software, Inc., San Diego, CA, USA). The data are expressed as mean ± standard deviation (SD) of three independent experiments. * *p* < 0.05 value denoted statistical significance. The Newman–Keuls method was utilized as a post hoc ANOVA assessment in the analysis of variance (ANOVA) for multiple groups, while the Student’s *t*-test was employed for the comparative study of two groups.

## 5. Conclusions

In conclusion, the present study revealed that 8PG is a novel AMPK activator from CGJ and is associated with inhibiting lipid accumulation and enhancing β-oxidation through the SIRT1-mediated pathway, thereby attenuating hepatic steatosis. Therefore, 8PG from CGJ could be an excellent therapeutic agent for combating hepatic steatosis, providing a new avenue for dietary intervention to treat MASLD; however, further in vivo experiments are warranted.

## Figures and Tables

**Table 1:** In silico docking simulation of the active metabolite, 8PG, from fermented soybean CGJ with AMPK.

Compound	AMPK (PDB ID: 2Y94)		
	AutoDock Vina	AutoDock4	LeDock
AICAR (Control)	−6	−7.54	−6.01
8PG	−8.7	−9.58	−5.02
Commonly shared residues	LEU22, GLY25, VAL30, VAL 96, LEU146, and ASP 157		

8PG, 8-prenylgenistein; AICAR, 5-aminoimidazole-4-carboxamide ribonucleotide; AMPK, adenosine 5′ monophosphate-activated protein kinase; ASP, aspartic acid; GLY, Glycine; LEU, leucine; VAL, valine. Data on commonly shared residues were obtained from AutoDock 4 through pharmacophore analysis.

**Table 2:** In silico docking simulation of the active metabolite, 8PG, from fermented soybean CGJ with SIRT1.

Compound	SIRT1 (PDB ID: 4I5I)		
	AutoDock Vina	AutoDock4	LeDock
Resveratrol (Control)	−8.5	−7.69	−4.89
8PG	−10.3	−11.11	−5.39
Commonly shared residues	GLY25, VAL30, VAL96, LEU146, and ASP157		

8PG, 8-prenylgenistein; ASP, Aspartic acid; GLY, Glycine; SIRT1, sirtuin 1; LEU, leucine; VAL, valine. Data on commonly shared residues were obtained from AutoDock 4 through pharmacophore analysis.

**Table 3:** Detailed comparison of the ADMET properties of 8PG with those of the control compounds for AMPK and SIRT1.

**Properties**	**AICAR**	**Resveratrol**	**8PG**
**Absorption**			
Human intestinal absorption (HIA %)	18.271181	88.479404	90.773093
Caco-2 cell Permeability (nm s^−1^)	6.79648	5.19172	10.5061
MDCK cell permeability (nm s^−1^)	0.583302	76.7444	0.188383
Skin permeability (logKp, cm h^−1^)	−5.17298	−3.15256	−2.95147
**Distribution**			
Plasma protein binding (%)	5.116658	100	98.929110
Blood–brain barrier penetration (Cb/Cb)	0.627516	1.73812	0.856453
**Metabolism**			
CYP2C19 inhibition	Non	Inhibitor	Inhibitor
CYP2C9 inhibition	Non	Inhibitor	Inhibitor
CYP2D6 inhibition	Non	Non	Non
CYP2D6 substrate	Non	Non	Non
CYP3A4 inhibition	Inhibitor	Inhibitor	Inhibitor
CYP3A4 substrate	Weakly	Non	Non
**Excretion**			
P-GP inhibition	Non	Non	Inhibitor
**Toxicity**			
Ames test	Mutagen	Mutagen	Mutagen
Carcino_rat	Non	Carcinogen	Non

Data were analyzed and obtained using the PreADMET tool. Green, positive; yellow, weak; red, negative; AICAR, 5-aminoimidazole-4-carboxamide ribonucleotide; 8PG, 8-prenylgenistein; CYP, cytochrome P450; P-GP, permeability glycoprotein; SIRT1, sirtuin 1; and AMPK, adenosine 5′ monophosphate-activated protein kinase.

## Data Availability

Data supporting the findings of this study are available from the corresponding author upon reasonable request. Some data may not be available owing to privacy or ethical restrictions.

## Supplementary Materials

The following supporting information can be downloaded at: https://www.mdpi.com/article/10.3390/ijms25179730/s1.
