# Supplementary material for: 8-Prenylgenistein Isoflavone in Cheonggukjang Acts as a Novel AMPK Activator Attenuating Hepatic Steatosis by Enhancing the SIRT1-Mediated Pathway
Source: Int J Mol Sci. 2024 Sep 8;25(17):9730. doi: 10.3390/ijms25179730 (PMC11395689; doi:10.3390/ijms25179730)
Supplement: Supplementary file 1 [file ijms-25-09730-s001.zip › ijms-3106248-supplementary.pdf]

Supplementary Table S1. List of high-content metabolites in Cheonggukjang (CGJ)

Sl.No	Classification	Metabolites
1		Malonylgenistin
2		Malonyldaidzin
3		Daidzein-7-O-phosphate
4		Daidzein-7-O-β-D-glucoside
5		8-Prenylgenistein
6	Isoflavone	6-carboxymethyl genistein
7		Genistin
8		Daidzein
9		Glycitein
10		Genistein
11		LysoPE16:0
12		LysoPE18:1
13	Lysophospholipids	LysoPC18:3
14		LysoPC18:2
15		LysoPC18:1
16		Dehydrosoyasaponin I
17		Soyasaponin I
18		Soyasaponin II
19	Soyasaponins	Soyasaponin III
20		Soyasaponin IV
21		Soyasaponin V
22		Pinitol
23	Sugar and sugar alcohols	N-Acetyl-D-Glucosamine
24		Sucrose
25		Aceticacid
26		Gluconicacid
27		Fumaricacid
28	Organic acids	Ribonicacid

29		Lacticacid
30		Glutaricacid
31		Benzoicacid
32	Fatty acids	Glyceryl Palmitate
33	Amino acids	Proline
34		Hydroxylamine

Supplementary Table S2. Detailed comparison of the ADMET properties of six isoflavones from CGJ

Properties	Malonylgenistin	malonyldaidzin	Daidzein7Oβ glucoside	Daidzein7Oβ phosphate	8PG	6 Carboxymethyl genistein
**Absorbtion**						
Human intestinal absorption (HIA %)	21.375782	41.952215	69.578674	79.174277	90.773093	77.349390
Caco-2 cell Permeability (nm s−1)	11.5585	13.1625	8.96177	4.60808	10.5061	9.39678
MDCK cell permeability (nm s−1)	0.596036	0.63324	1.51434	3.00322	0.188383	30.3626
Skin permeability (logKp, cm h−1)	-4.11764	-4.03992	-4.51605	-1.86792	-2.95147	-4.28932
Plasma Protein Binding (%)	65.096902	68.822937	67.953147	100	98.929110	82.313118
Blood–brain barrier penetration	0.0256474	0.0248615	0.036872	0.00923007	0.856453	0.0222978
**Metabolism**						
CYP2C19 inhibition	inhibitor	inhibitor	inhibitor	Non	inhibitor	inhibitor
CYP2C9 inhibition	inhibitor	inhibitor	inhibitor	inhibitor	inhibitor	inhibitor
CYP2D6 inhibition	Non	Non	Non	Non	Non	Non
CYP2D6 Substrate	Non	Non	Non	Non	Non	Non
CYP3A4 inhibition	inhibitor	inhibitor	Non	Non	inhibitor	inhibitor
CYP3A4 Substrate	Weakly	Weakly	Weakly	Weakly	Non	Non
**Excretion**						
Pgp inhibition	Non	Non	Non	inhibitor	inhibitor	Non
**Toxicity**						

Ames_test	Mutagen	Mutagen	Mutagen	Mutagen	Mutagen	Mutagen
Carcino_Mouse	Positive	Positive	Positive	Positive	Negative	Negative
Carcino_Rat	Negative	Negative	Negative	Negative	Positive	Positive

Data were analyzed and obtained using the PreADMET tool. Green, positive; yellow, weak; red, negative; 8PG, 8-prenylgenistein; CYP, cytochrome P450; and P-GP, permeability glycoprotein.

Supplementary Table S3. *In silico* docking simulation of the Genistein and 8PG with AMPK

		AMPK (PDB id: 2Y94)		
**Sl.no**	**Compounds**	**Autodock Vina**	**AutoDock4**	**LeDock**
1	AICAR (Control)	-6	-7.54	-6.01
2	Genistein	-7.7	-8.15	-4.18
**3**	**8- Prenylgenistein**	**-8.7**	**-9.58**	**-5.02**

Supplementary Table S4. *In silico* docking simulation of the active metabolites from fermented soybean CGJ with AMPK and SIRT1

		AMPK (PDB id: 2Y94)			SIRT1 (PDB id:4I5I)		
**Sl. no**	**Compounds**	**Autodock Vina**	**AutoDock4**	**Dock6**	**Autodock Vina**	**Auto Dock4**	**Dock6**
1	Positive control	-5.7	-10.81	-132.631	-7.9	-9.23	-38.382
2	Malonylgenistin	-8	-10.27	-46.6365	-7.7	-11.7	-44.7166
3	Malonyldaidzin	-7.9	-11.38	-43.1116	-7.6	-11.12	-42.8477
4	Daidzein-7Oβ glucoside	-8.2	-10.77	-42.0299	-8.4	-12.07	-42.6161
5	Daidzein-7Oβ phosphate	-7.9	-7.08	-34.8626	-8.2	-8.78	-36.7709
6	**8-Preneyilgenistein**	**-8.7**	**-9.58**	**-36.6164**	**-10.3**	**-11.11**	**-40.4357**
7	6 Carboxymethylgenistein	-8.7	-7.81	-36.1463	-9.1	-10.58	-40.535
